# Assessment of the inferior vena cava collapsibility from subcostal and trans-hepatic imaging using both M-mode or artificial intelligence: a prospective study on healthy volunteers

**DOI:** 10.1186/s40635-023-00505-7

**Published:** 2023-04-03

**Authors:** Filippo Sanfilippo, Luigi La Via, Veronica Dezio, Cristina Santonocito, Paolo Amelio, Giulio Genoese, Marinella Astuto, Alberto Noto

**Affiliations:** 1 Department of Anaesthesia and Intensive Care, A.O.U. Policlinico-San Marco, site “Policlinico G. Rodolico”, Via S. Sofia N 78, 95123 Catania, Italy; 2 School of Anaesthesia and Intensive Care, University Hospital “G. Rodolico”, University of Catania, 95123 Catania, Italy; 3 Division of Anesthesia and Intensive Care, University of Messina, Policlinico “G. Martino”, Messina, Italy; 4 Department of Human Pathology of the Adult and Evolutive Age “Gaetano Barresi”, Division of Anesthesia and Intensive Care, University of Messina, Policlinico “G. Martino”, Messina, Italy

**Keywords:** Critical care, Ultrasound, Subcostal, Transhepatic, Inferior vena cava

## Abstract

**Purpose:**

Assessment of the inferior vena cava (IVC) respiratory variation may be clinically useful for the estimation of fluid-responsiveness and venous congestion; however, imaging from subcostal (SC, sagittal) region is not always feasible. It is unclear if coronal trans-hepatic (TH) IVC imaging provides interchangeable results. The use of artificial intelligence (AI) with automated border tracking may be helpful as part of point-of-care ultrasound but it needs validation.

**Methods:**

Prospective observational study conducted in spontaneously breathing healthy volunteers with assessment of IVC collapsibility (IVCc) in SC and TH imaging, with measures taken in M-mode or with AI software. We calculated mean bias and limits of agreement (LoA), and the intra-class correlation (ICC) coefficient with their 95% confidence intervals.

**Results:**

Sixty volunteers were included; IVC was not visualized in five of them (*n* = 2, both SC and TH windows, 3.3%; *n* = 3 in TH approach, 5%). Compared with M-mode, AI showed good accuracy both for SC (IVCc: bias − 0.7%, LoA [− 24.9; 23.6]) and TH approach (IVCc: bias 3.7%, LoA [− 14.9; 22.3]). The ICC coefficients showed moderate reliability: 0.57 [0.36; 0.73] in SC, and 0.72 [0.55; 0.83] in TH. Comparing anatomical sites (SC vs TH), results produced by M-mode were not interchangeable (IVCc: bias 13.9%, LoA [− 18.1; 45.8]). When this evaluation was performed with AI, such difference became smaller: IVCc bias 7.7%, LoA [− 19.2; 34.6]. The correlation between SC and TH assessments was poor for M-mode (ICC = 0.08 [− 0.18; 0.34]) while moderate for AI (ICC = 0.69 [0.52; 0.81]).

**Conclusions:**

The use of AI shows good accuracy when compared with the traditional M-mode IVC assessment, both for SC and TH imaging. Although AI reduces differences between sagittal and coronal IVC measurements, results from these sites are not interchangeable.

## Introduction

Assessment of fluid responsiveness (FR) is challenging but at the same time crucial for the correct management of patients admitted to intensive care unit (ICU); indeed conditions of reduced or increased volemia are both associated with worse clinical outcomes [1]. Hypovolemia and the consequent reduction in preload decreases stroke volume with the risk of organ hypo-perfusion [2], while presence of hypervolemia and fluid overload may determine congestion and edema, in turn impairing organ perfusion [3–5]. Therefore, assessment of FR is usually needed multiple times a day in ICU patients [6] as they have significant variability in loading conditions according to different variables (fluctuations in sedation level, changes in vasomotor tone [7], modifications in capillary leakage according to both inflammation and infections [8, 9], etc.).

Several methods are available for the prediction of FR, each one with different sensibility and specificity in predicting an increase in cardiac output or stroke volume after fluid administration [10, 11]. Some of these methods are invasive and requires arterial cannulation and eventually advanced cardiac output monitoring, while other tools are non-invasive. Among the latter ones, the estimation of inferior vena cava (IVC) diameter variations with echocardiography is probably the most widely studied. Prediction of FR using the IVC variation has been validated for both mechanically ventilated patients (IVC distensibility, IVCd = ΔIVC/IVC minimum diameter) with a cutoff of 18% [12], and for spontaneously breathing patients (IVC collapsibility, IVCc = ΔIVC/IVC maximum diameter) with variable cutoffs in the range of 40–48% [13–15]. Although the reliance on IVC respiratory variation has several limitations [16–18], the interest around this parameter is explained by its relatively high feasibility in most critically ill patients [19, 20]. Nonetheless, IVC assessment with standard subcostal approach (SC or sagittal) is not always feasible due to enlarged bowel, obesity, presence of chest drains or laparotomy wounds. In such cases, an alternative approach could be represented by the assessment of the IVC with trans-hepatic approach (TH or coronal, or right lateral), with a latero-lateral visualization of the vessel. However, there are conflicting reports on the reliability of IVC assessment using TH approach [21, 22]: moreover, a recent systematic review showed that there is limited evidence regarding the interchangeability of TH and SC views. In particular, only seven studies were identified and these studies have shown gross heterogeneity in their design and participants enrolled (ventilated, spontaneously breathing, or mixed populations), as well as in the approaches for data reporting and analysis. Therefore, the systematic review suggested that more research is warranted, with high likelihood of discordance between SC and TH approaches due to inhomogeneous respiratory variation in IVC shape, with IVC collapse happening more commonly in the antero-posterior direction [23].

The use of artificial intelligence (AI) is in great expansion in several medical fields, including echocardiography. For instance, AI has been applied to the estimation of left ventricular systolic [24, 25] and diastolic [26–28] function, to right ventricular function [29], but it has also been adopted for assessment of heart valve diseases [30, 31] and for diagnosis of congenital heart diseases [32]. Furthermore, machine learning methods have been developed for the improvement of bedside prediction of FR [33], and preliminary experiences with AI in the assessment of IVCc have been reported [34].

In consideration of: (1) the heterogeneity in the studies evaluating the differences in IVC size and variation with SC and TH approach, and (2) the uncertainty on whether the use of AI method may improve (or not) accuracy and precision of this parameter, we conducted a prospective study to answer these questions.

## Materials and methods

We designed a prospective observational study in healthy volunteers, followed by a study in mechanically ventilated patients. We aimed at evaluating differences in SC and TH visualization of the IVC, performed by an experienced operator in M-Mode method or by an AI software. In this manuscript we report the data regarding the spontaneously breathing healthy volunteers, which were recruited from personnel of the School of Anaesthesia and Intensive Care of the University of Catania and from the staff of the General ICU of the *Azienda Ospedaliera Universitaria “Policlinico-San Marco”*, Catania. The study was approved from our local Ethical Committee (Reference protocol: 53/2022/PO).

### Participants 

We included healthy adult volunteers recruited from the staff of our department, regardless of their age and gender. Participants were instructed to breath normally during the examination. Exclusion criteria were missing informed consent.

### Study interventions 

Echocardiography was performed by a single experienced operator (FS) using a portable machine *General Electric (GE) Venue Go R2* equipped with AI software for automated border tracking of the IVC. The volunteers were positioned in a bed in semi-recumbent (30°–35°) position. The operator recorded several imaging of the IVC in both SC and TH approach. In particular, images were recorded with two methods, the M-mode and the AI ones. The M-mode provides imaging (single line) at very high frame as compared to 2-D echocardiography, allowing accurate determination of linear dimensions and improving quantitation of sizes (vessels, chambers, wall thickness, etc.). For the purpose of the study, M-mode imaging was recorded by the operator for a subsequent offline calculation of the IVC diameters and of the IVCc index. The second method of image acquisition for the study is based on AI, and in particular on the use of the software for automated border tracking adapted to the IVC. Following image recording with M-mode, the operator performed repeated measures with this automated border detection function (each clip lasting 6 s), and all were included for analysis.

### Study groups and outcomes

From the combination of the site of image acquisition (SC or TH) and the modality of data calculation, four groups of data were generated: (1) SC in M-mode; (2) SC in AI; (3) TH in M-mode; (4) TH in AI. Our study had a factorial 2 × 2 design, focusing on the differences and correlations of IVC measurements according to:

A. *Different acquisition modality****:*** the same site of acquisition but with different measuring modality (M-mode vs AI), thus comparing:

- SC-in M-mode vs SC in AI; and
- TH in M-mode vs TH in AI;

B. *Different acquisition site:* the same measuring modality with different site of imaging (SC vs TH), thus comparing:

- SC in M-mode vs TH in M-mode; and
- SC in AI vs TH in AI.

The variable of primary interest in our study was the IVCc index. As secondary endpoints we analyzed the IVC diameters (IVC maximum and IVC minimum).

### Statistical analysis 

Two previous studies reported mean values of IVC diameters in spontaneously breathing volunteers for both SC and TH view [35, 36]; in these studies, the IVCc had a range between 42% and 58%, and the IVCc differences between SC and TH view were comprised in the range 7% [35] to 15% [36]. However, standard deviation was not available for the IVCc. With such premise, to avoid the risk of an underpowered study, we calculated the sample size considering a much more conservative value for the mean IVCc index of 30 ± 5% with an estimated mean difference between SC and TH of 4%. Thus, assuming a statistical power of 80% and an α level of significance at 0.05, the sample size calculation suggested enrolling 50 healthy volunteers. Considering up to a maximum of 20% for missing views in TH or SC *(n* = 10), we planned to enroll 60 adult healthy volunteers.

We calculated the agreements mean bias, and limits of agreement [LoA] between IVC measurements in different areas/modalities with the Bland and Altman plots. Bland–Altman plots and statistics were adjusted for the effect of multiple measures as described by Zou only for the comparison of AI modalities [37]. The bias indicates the accuracy of measurements methods, while the LoA specifies the precision. Their values are reported with the relative 95% confidence interval. Considering that the best FR cutoff using the IVCc index in spontaneously breathing patients has been reported in the range of 40–48% [13–15], we decided that a mean bias of 8% and 4% would describe acceptable and good accuracy, respectively. Regarding the precision (LoA) of the measurements, we considered a range of 32% and 16% as acceptable and good precision, respectively.

The relationship among variables was evaluated calculating the intra-class correlation (ICC) coefficient to describe the inter-rater variability between measures acquired with the same modality (AI TH vs AI SC, or M-mode TH vs M-mode SC) or in the same approach (AI TH vs M-mode TH, or AI SC vs M-mode SC) resemble each other. Interpretation of correlation was performed according to established cutoffs[38].

## Results

Descriptive statistics of the volunteers participating in the study are reported in Table 1. Of the 60 volunteers included, two patients did not have both SC and TH windows (3.3%), and for further three (5%) it was not possible to obtain the TH visualization. Hence, full data were collected for 55 volunteers. The mean IVCc index in SC imaging was 33.3 ± 12.6%, while it was much lower for the TH imaging (19.7% ± 11.5).

**Table 1:** Baseline characteristics of included volunteers

Baseline characteristics (*n* = 60)		Average Measurements in M-mode (*n* = 55)	
Gender (male)	33/60	IVCmin in SC (mm)	14.8 ± 4.4
Age (years)	32.1 ± 8.6	IVCmax in SC (mm)	21.9 ± 4.2
Weight (Kg)	71.1 ± 15.7	IVCc in SC (%)	33.3 ± 12.6
Height (cm)	171.4 ± 8.5	IVCmin in TH (mm)	19.2 ± 4.9
BMI (kg/m^2^)	24.0 ± 4.0	IVCmax in TH (mm)	23.7 ± 4.2
		IVCc in TH (%)	19.7 ± 11.5

All the results of the Bland Altman plots are reported in Table 2, where the mean bias, the lower and the upper LoA with their 95%CI are shown. In the same table we report also the intraclass correlation coefficient to describe how strong measurements resemble each other.

**Table 2:** Summary of comparisons made between measurement of the inferior vena cava (IVC) in healthy volunteers

Comparison		Variable	Mean Bias and 95% CI	LoA and 95% CI		ICC and 95% CI
				Lower	Upper	
AI-SC	M-SC	IVCc	− 0.7% [− 4.1 to 2.7]	− 24.9% [− 30.8 to v19.1]	23.6% [17.7 to 29.5]	0.57 [0.36 to 0.73]
		IVC-max	− 2.5 mm [− 3.1 to − 2.0]	− 6.7 mm [− 7.7 to − 5.7]	1.6 mm [0.6 to 2.6]	0.83 [0.73 to 0.90]
		IVC-min	− 1.4 mm [− 2.1 to − 0.7]	− 6.3 mm [− 7.5 to − 5.1]	3.5 mm [2.4 to 4.7]	0.81 [0.69 to 0.89]
AI-TH	M-TH	IVCc	3.7% [1.0 to 6.3]	− 14.9% [− 19.5 to − 10.3]	22.3% [17.7 to 26.8]	0.72 [0.55 to 0.83]
		IVC-max	− 2.4 mm [− 3.2 to − 1.5]	− 8.1 mm [− 9.5 to − 6.7]	3.4 mm [1.9 to 4.8]	0.67 [0.49 to 0.80]
		IVC-min	− 2.6 mm [− 3.4 to − 1.8]	− 8.3 mm [− 9.7 to − 6.9]	3.0 mm [1.6 to 4.4]	0.79 [0.66 to 0.87]
M-SC	M-TH	IVCc	13.9% [9.5 to 18.3]	− 18.1% [− 25.7 to − 10.5]	45.8% [38.3 to 53.4]	0.08 [− 0.18 to 0.34]
		IVC-max	− 1.7 mm [2.8 to − 0.6]	− 9.6 mm [− 11.5 to − 7.7]	6.1 mm [4.3 to 8.0]	0.53 [0.32 to 0.70]
		IVC-min	− 4.4 mm [− 5.8 to − 3.0]	− 14.5 mm [− 16.9 to − 12.1]	5.6 mm [3.2 to 8.0]	0.38 [0.13 to 0.59]
AI-SC	AI-TH	IVCc	7.7%	− 19.2% [− 24.6 to − 15.1]	34.6% [30.4 to 40.0]	0.69 [0.52 to 0.81]
		IVC-max	− 2.0 mm	− 9.2 mm [− 10.9 to − 7.9]	5.2 mm [3.9 to 6.9]	0.45 [0.21 to 0.65]
		IVC-min	− 2.9 mm	− 10.0 mm [− 11.6 to − 8.8]	4.3 mm [3.1 to 5.9]	0.67 [0.49 to 0.80]

In case of the IVC size analysis in M-mode (M), we analyzed a single measure which was the most reliable measure as decided by the experienced operator performing the calculations. In case of the analysis with artificial intelligence (AI), repeated measures were taken and saved in the database. Results of IVC collapsibility, minimum and maximum diameters (IVCc, IVC-min and IVC-max, respectively) are provided in terms of mean Bias and limits of agreement (LoA) with their relative 95% confidence interval (CI), where appropriate. We also provide intraclass correlation coefficient (ICC) to describe how strong the measurements resemble each other

### Different acquisition modality

**Fig. 1:**
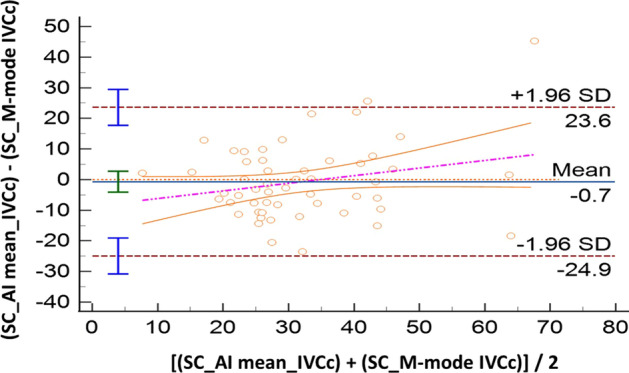
Bland–Altman plot for the inferior vena cava collapsibility index (IVCc) measured in subcostal (SC) site with standard M-mode or artificial intelligence (AI). SD: standard deviation

**Fig. 2:**
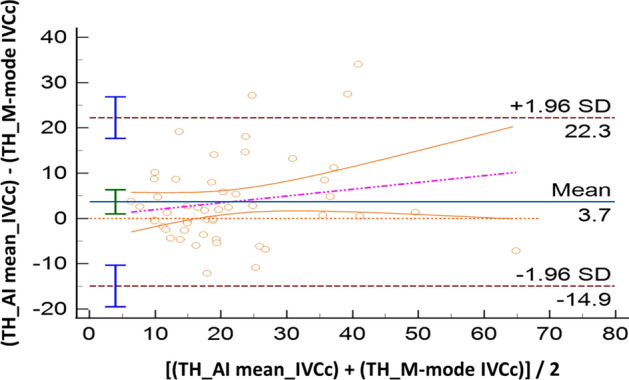
Bland–Altman plot for the inferior vena cava collapsibility index (IVCc) measured in Transhepatic (TH) site with standard M-mode or artificial intelligence (AI). SD: standard deviation

Comparing AI and M-mode strategy for IVC assessment, measurements, where similar both for SC IVCc (bias -0.7%, LoA [− 24.9; 23.6], Fig. 1) and diameters (IVC maximum: bias − 2.5 mm, LoA [− 6.7; 1.6]; IVC minimum: bias − 1.4 mm, LoA [− 6.3; 3.5]), as well as for the TH IVCc (bias 3.7%, LoA [− 14.9; 22.3]; Fig. 2) and diameters (IVCmaximum: bias − 2.4 mm, LoA [− 8.1; 3.4]; IVC minimum: bias − 2.6 mm, LoA [− 8.3, 3.0]).

Overall, the ICC coefficients showed moderate reliability; in particular, the ICC for the IVCc was 0.57 [0.36, 0.73] for SC and 0.72 [0.55, 0.83] for TH.

### Different acquisition site

**Fig. 3:**
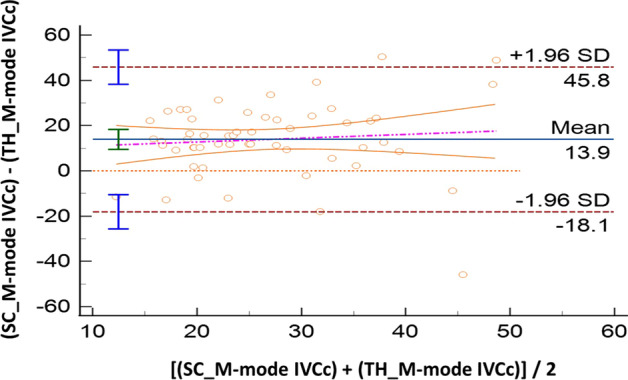
Bland–Altman plot for the inferior vena cava collapsibility index (IVCc) measured with standard M-mode in two different sites: subcostal (SC) and transhepatic (TH). SD: standard deviation

**Fig. 4:**
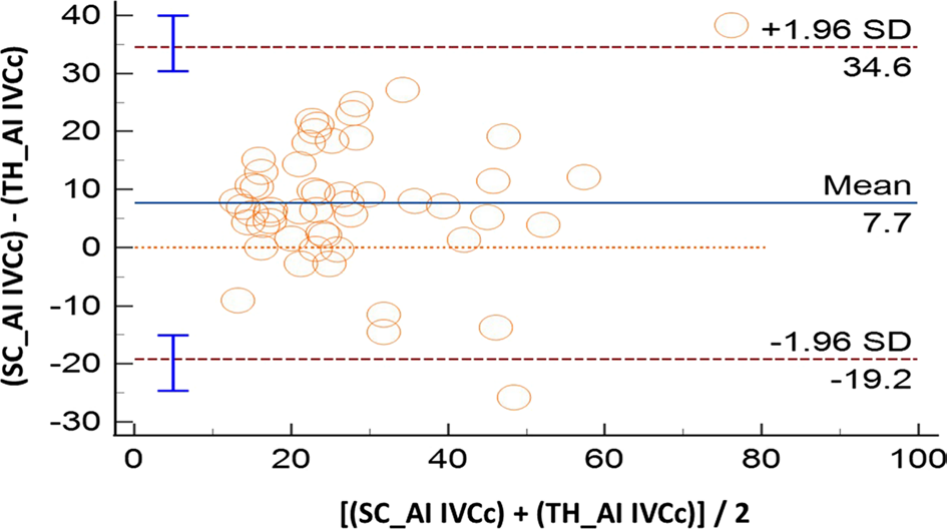
Bland–Altman plot for the inferior vena cava collapsibility index (IVCc) measured with artificial intelligence (AI) mode in two different sites: subcostal (SC) and transhepatic (TH). SD: standard deviation

When the assessments of the IVC were compared between anatomical sites (SC vs TH) we found that results are not clinically interchangeable. In particular, comparing the SC and TH M-mode assessment, IVCc had a mean bias 13.9% with LoA [− 18.1; 45.8] (Fig. 3); also, the IVC diameters showed differences between anatomical sites (IVC maximum: bias − 1.7 mm, LoA [− 9.6; 6.1]; IVC minimum: bias − 4.4 mm, LoA [− 14.5; 5.6]). When the evaluation was performed with the aid of AI, the differences between SC and TH seemed smaller but still of likely clinical impact for the IVCc (bias 7.7%, LoA [− 19.2; 34.6]; Fig. 4) and the diameters (IVC maximum: bias − 2.0 mm, LoA [− 9.2; 5.2]; IVC minimum: bias -2.9 mm, LoA [− 10.0, 4.3]).

With the exception of the IVCc in M-mode that showed non-significant correlation (ICC = 0.08 [− 0.18, 0.34]), the other ICC coefficients comparing SC and TH measurements showed significant correlations but of moderate or mild degree (r range between 0.38 and 0.69; Table 2). In particular, the ICC for the IVCc calculated with AI in SC and TH view was 0.69 [0.52, 0.81].

## Discussion

Our investigation focused on the assessment of the IVCc (and of its diameters) collected at two different sites (sagittal SC, and coronal TH) with different measuring modality (M-Mode vs AI). Our study resembles a 2 × 2 factorial design, intertwining evaluations of the IVC from different technical and anatomical perspectives. The main findings of this study conducted on healthy volunteers support the clinical introduction of AI for the estimation of IVC size at the bedspace while does not suggest an interchangeable use of the results gathered from SC and TH imaging. The discussion of these findings is divided in the one on the clinical role of AI in IVC assessment, and then in the differences between imaging in sagittal or coronal approach.

Looking at the role of the automated border detection (AI) for the IVC assessment, which is the more original and innovative point of the study, our results suggest that the introduction of AI may be great help for clinicians. Indeed, considering the accuracy of this method, AI has the potentialities of saving time for bedside assessment of FR. Indeed, from practical perspectives, the use of M-Mode method requires image acquisition and freeze, and then calculation of the IVC diameters and application of a formula. Conversely, use of automated border detection (AI function) abolishes three steps: freeze of the image, calculation of the IVC diameters, and application of formula; hence, AI allows real-time calculation just simply obtaining the image and holding the probe in place. The time saved may allow clinicians to perform a greater number of calculations of IVCc (or IVCd, according to the ventilation mode), which could be averaged especially in case of borderline results. Furthermore, with the help of automated border detection the operator can hold his/her hand on the probe and watch the screen, while the machine keeps calculating values of IVCc (or IVCd). In this regard, we found very good accuracy for the AI calculation as compared to the standard M-mode approach, with a bias of − 0.7% (good accuracy) for the SC view and of 3.7% for the TH imaging. However, it must be noted that in both cases the LoA were relatively wide, indicating low precision. Nonetheless, as shown by the violet dotted lines in the Bland–Altman plots (Figs. 1 and 2), in both cases (SC and TH) there was a clear trend in such dispersion, which was greater for the higher IVCc values (over ~ 30% for the SC, and over ~ 20% for the TH). This finding derives probably from the challenge in estimating the IVC when it is almost fully collapsible, with the IVC minimum becoming lower than 0.5 mm. In such cases, the evaluation in M-mode using the touch screen (as for the machine used for our study) may be prone to small errors, and the LoA (precision) could be narrower when approaching mechanically ventilated patients with distended IVC. As mentioned, machine learning generated models have been developed for the prediction of FR[33], with encouraging (comparable or superior) results when compared to the hemodynamic response to passive leg raising. Blaivas et al. conducted pioneer studies on this topic using deep learning algorithm capable of video classification for the estimation of FR with the IVC imaging; the authors showed that the trained deep learning algorithm performed moderately well (area under the curve 0.70; 95% CI 0.43–1.00) [34]. Moreover, the same group of authors showed that performances of this validated deep learning algorithm were dependent on the image quality (much worse on images from a lower quality device) [39]. In summary, the findings of our study on the value of introducing AI for the calculation of the IVC indexes of FR seem encouraging, and may be supportive of its introduction in clinical practice. However, it is of utmost importance to validate these findings in populations of mechanically ventilated patients, where theoretically, the results could be even more interchangeable due to the greater vessel size with potentially larger agreement between AI and standard M-mode measurements.

Regarding the second part of the study, results of IVCc obtained in SC were compared with those recorded in TH approach. As suggested by a recent systematic review that included seven studies in different cohorts of volunteers or patients (spontaneously breathing, mechanically ventilated, hybrid) [23], the results of SC and TH imaging for IVCc may be significantly different and not truly interchangeable, with IVC variation that seems usually greater in the antero-posterior direction rather than in the latero-lateral axis. Our study points in the same direction as we found a mean bias of ~ 14% for the M-mode assessment of the IVCc taken at the two different anatomical sites; moreover, we found significant dispersion with a very large LoA range of ~ 64%. In such case, there was not a clear trend, indicating that dispersion and differences is constant in all ranges of IVCc estimates. In concordance with the above-mentioned systematic review [23], the present results suggest that IVCc obtained in M-mode from SC view is greater than the M-mode finding gathered with TH approach. Therefore, it seems that the IVC collapses more in antero-posterior direction rather than in the latero-lateral one. Surprisingly, the use of AI in comparing results for SC and TH imaging showed with borderline bias (~ 8%), but still with a low precision (LoA range ~ 54%). However, these results should not discourage research for the introduction of cutoffs for the prediction of FR with the IVC imaged in coronal view (TH); conversely these findings should foster investigations looking at the best cutoff in predicting FR when using TH-derived parameters. Importantly, the use of TH IVCc can be very valuable in patients, where the imaging in the SC region is not achievable for clinical reasons as obesity, or for the presence of laparotomy wound or mediastinal drains. It must be kept in mind that the TH imaging is not always feasible and in our population of healthy volunteers, five out of 60 (8.3%) did not have a TH view (and two of them did not have the SC either).

### Strengths and limitations

The main strength of our study was the originality in investigating with AI method the differences in SC and TH imaging. In addition, our study was conducted in a homogeneous population of healthy and young volunteers. Moreover, considering the mean results for IVCc index in SC and TH imaging (33.3% ± 12.6 and 19.7% ± 11.5, respectively) a post hoc sample size calculation showed that 26 volunteers would have been appropriate, therefore, suggesting that the study was appropriately powered.

Our study has several limitations. First, although the sample size was bigger than previous studies [35, 36] and considering the results our study seems well-powered, it was conducted in healthy and relatively young volunteers; therefore, its results could be different in spontaneously breathing patients that could be older than our population and are likely to present associate comorbidities. Second, a single experienced operator collected the images and performed the M-mode calculations, and results may be different in less experienced hands. Third, we did not assess the inter-observer variability. Fourth, the image acquisition followed a schematic pattern to avoid mistakes but an ideal study design would have provided randomization for the order of image acquisition. Nonetheless, we believe this is unlikely to influence results but it remains fair to acknowledge such item. Finally, it must be reminded that several limitations exist for the clinical application of IVC indexes of FR, especially when referring to mechanically ventilated patients that are usually treated with protective ventilation criteria. Therefore, independently from the potentialities of the implementation of AI method, clinicians should be always aware of the limitations of each method for assessment of FR.

### Future perspectives

Considering the results of our study conducted on healthy volunteers, it seems appropriate to perform clinical studies on mechanically ventilated patients admitted to ICU. In this regard, research on the value of adding AI with automated border detection for estimation of FR using the IVC is warranted, both in terms of accuracy of measurements and in respect to the clinical advantages in terms of time saved, which in turn could be devoted to multiple calculation or to other relevant tasks. Moreover, further research may confirm that the use of TH view does not seem interchangeable with the data gathered from the SC imaging. Nonetheless, it remains to be established whether, with the calculation and adoption of different cutoffs for FR, the use of TH visualization of IVC may be add valuable information at the bed-space.

## Conclusions

The use of artificial intelligence for the assessment of the inferior vena cava collapsibility shows very good accuracy when compared with the traditional M-mode evaluation, both for subcostal and transhepatic imaging. The greater dispersion of the measurements is seen for the higher values of inferior vena cava collapsibility. Although adoption of artificial intelligence may reduce the differences between sagittal and coronal assessment, results from these two anatomical sites do not seem clinically interchangeable.

## Data Availability

On request to the corresponding author.

## Abbreviations

AI: Artificial intelligence
CI: Confidence interval
FR: Fluid responsiveness
IVC: Inferior vena cava
IVCc: IVC collapsibility
IVCd: IVC distensibility
ICU: Intensive care unit
ICC: Intra-class correlation
LoA: Limits of agreement
SC: Subcostal
TH: Trans-hepatic
